# Finding FISH in a small pond

**DOI:** 10.7554/eLife.16598

**Published:** 2016-05-20

**Authors:** Calvin H Jan

**Affiliations:** Calico Life Sciences LLC, South San Francisco, United Statescjan@calicolabs.com

**Keywords:** transcriptome, superresolution microscopy, fluorescence in situ hybridization, RNA degradation, spatial organization, next-generation sequencing, *E. coli*

## Abstract

Advanced microscopy and labeling techniques reveal that bacteria localize mRNAs within their cells in a similar way to eukaryotes.

**Related research article** Moffitt JR, Pandey S, Boettiger AN, Wang S, Zhuang X. 2016. Spatial organization shapes the turnover of a bacterial transcriptome. *eLife* **5**:e13065. doi: 10.7554/eLife.13065

**Image** ‘FISH’ probes show where mRNAs reside in *E. coli* cells

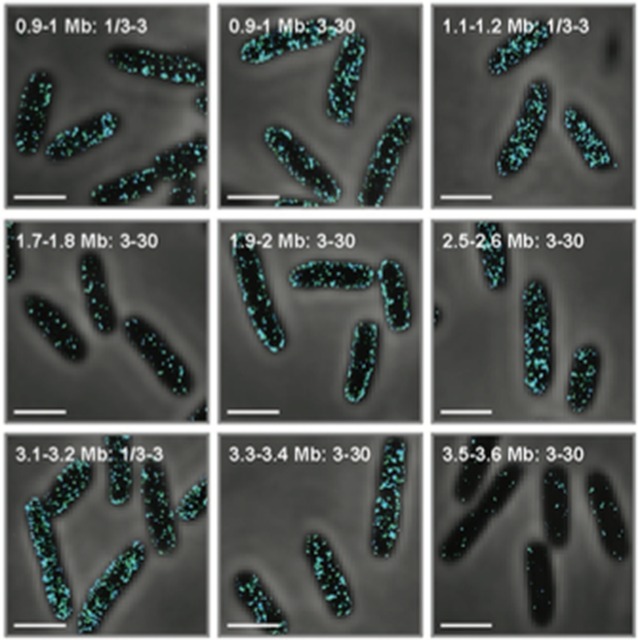

It makes sense to organize manufacturing around infrastructure. For example, if you are building ships, it is better to build them by the sea than inland. The major form of manufacturing in a cell is protein synthesis and, like shipbuilders, cells often make proteins at the sites where they are needed (Buxbaum et al., 2015). This is possible because mobile mRNA molecules can carry genetic information from the chromosomes to other sites in the cell.

Most genes in eukaryotes reside in the nucleus, but the mRNAs must be translated into proteins outside of the nucleus in the cytosol. It is thus understandable why most researchers interested in mRNA localization study the process in eukaryotic cells rather than in bacteria (which, of course, don’t have a nucleus). Bacterial cells are also, as a rule, smaller than those of eukaryotes. In fact, most bacteria are not much bigger than the diffraction limit of light. This means that it is difficult to distinguish between different structures within a bacterium under a conventional light microscope, which is likely another reason why we know much less about RNA localization in bacteria.

Now, in eLife, Xiaowei Zhuang and colleagues at Harvard University – including Jeffrey Moffitt as first author – report that the bacterium *Escherichia coli* also localizes its mRNAs within its cells (Moffitt et al., 2016). The Harvard team solved the problem related to the bacterium’s small size by combining a super-resolution microscopy technique called STORM (Huang et al., 2008) with a labeling technique called FISH (short for “fluorescent in situ hybridization”). Their labeling approach relied on engineering fluorescent probes that bind to specific subsets of mRNAs. These probes revealed that, like in eukaryotes, different mRNAs in bacterial cells are organized at different locations (Figure 1). Specifically, Moffitt et al. found that mRNAs that encode proteins destined for the inner membrane of the bacterium were enriched at this membrane.

**Figure 1.**
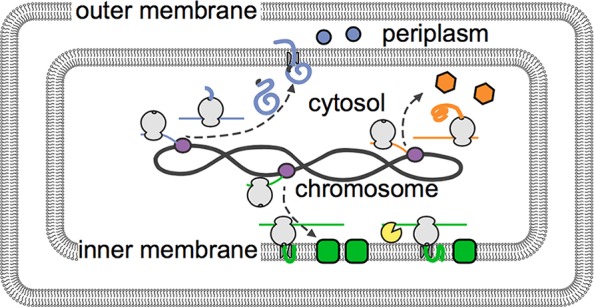
The distribution of mRNAs in *E. coli* reflects how newly built proteins are processed. Genetic instructions encoded in the DNA of the chromosome are copied, or transcribed, by enzymes called RNA polymerases (purple) into mRNAs (thin lines). These mRNA are then translated into proteins by ribosomes (depicted in gray). Moffitt et al. show that mRNAs that encode soluble proteins (orange) diffuse freely from the chromosome and are translated throughout the cytosol. Similarly, mRNAs that encode proteins that will be release into the periplasm or beyond (blue) are found throughout the cytosol. Membrane proteins (green) are insoluble in the cytosol and are instead translated at the inner membrane. The degradasome (yellow) is also found at the inner membrane and preferentially breaks down nearby mRNAs.

Most membrane proteins insert into membranes via hydrophobic (or ‘water-hating’) sections that sit within the oil-like interior of a membrane (Sharpe et al., 2010). However, these hydrophobic sections also make the proteins prone to aggregating in the cytosol, which is mostly water. Inserting the proteins into the membrane whilst they are being built neatly solves this problem.

A protein-RNA complex called the signal recognition particle (SRP) recognizes a hydrophobic portion of a membrane protein as it emerges from the ribosome (the cellular machine that translates mRNA molecules into proteins). The SRP then shields this part of the protein, which is referred to as a signal peptide, from the watery cytosol and brings it to the membrane. In eukaryotes, a specific part of the SRP temporarily pauses the ribosome until it reaches the membrane. The SRP in bacteria lacks this part (Halic et al., 2004), yet it can nevertheless interact with an active ribosome (Noriega et al., 2014). This suggested that the SRP might still work in a similar way in eukaryotes and bacteria.

Moffitt et al. decided to test this idea. They found that mRNAs for a synthetic protein with a signal peptide did indeed localize to the inner membrane of *E. coli*. However, this didn’t happen if an antibiotic called kasugumycin prevented the translation process from starting. Together, these data show that the signal peptide recruits mRNAs to the membrane.

A complex of enzymes that processes and degrades RNA also localizes to the inner membrane of *E. coil* (Mackie, 2013). This led Moffitt et al. to ask if membrane-localized mRNAs are less stable than their counterparts in the cytosol. The answer to this question is yes: on average, membrane-associated mRNAs have shorter lives than those in the cytosol. Inhibiting translation, which is required for localization to the membrane, counteracted this effect. Preventing the RNA-degrading enzyme complex from localizing to the membrane also protected the membrane-associated mRNAs. Further research is now required to understand the biological consequences of coupling membrane localization with enhanced breakdown of RNAs.

The Nobel prize-winning biologist Jacques Monod famously quipped, “what’s true of *E. coli* is true of the elephant, only more so.” Moffitt et al. have now once again proven Monod right. Both *E. coli* and elephants (as an example of a eukaryote) localize many mRNAs within their cells. Equipped with new and powerful techniques, it will be exciting to see what else elephants can teach us about bacteria and vice versa.
